# Modified Biomass-Reinforced Polylactic Acid Composites

**DOI:** 10.3390/ma17020336

**Published:** 2024-01-09

**Authors:** Junjie Zhu, Hui Sun, Biao Yang, Yunxuan Weng

**Affiliations:** 1 College of Light Industry Science and Engineering, Beijing Technology and Business University, Beijing 100048, China; z337616@163.com (J.Z.); ybiao@th.btbu.edu.cn (B.Y.); 2 Beijing Key Laboratory of Quality Evaluation Technology for Hygiene and Safety of Plastics, Beijing Technology and Business University, Beijing 100048, China

**Keywords:** polylactic acid (PLA), modified biomass, composite material

## Abstract

Polylactic acid (PLA), as a renewable and biodegradable green polymer material, is hailed as one of the most promising biopolymers capable of replacing petroleum-derived polymers for industrial applications. Nevertheless, its limited toughness, thermal stability, and barrier properties have restricted its extensive application. To address these drawbacks in PLA, research efforts have primarily focused on enhancing its properties through copolymerization, blending, and plasticization. Notably, the blending of modified biomass with PLA is expected not only to effectively improve its deficiencies but also to maintain its biodegradability, creating a fully green composite with substantial developmental prospects. This review provides a comprehensive overview of modified biomass-reinforced PLA, with an emphasis on the improvements in PLA’s mechanical properties, thermal stability, and barrier properties achieved through modified cellulose, lignin, and starch. At the end of the article, a brief exploration of plasma modification of biomass is presented and provides a promising outlook for the application of reinforced PLA composite materials in the future. This review provides valuable insights regarding the path towards enhancing PLA.

## 1. Introduction

With the development of the economy and the progress of society, there is a growing environmental awareness among people, making sustainable development an inevitable trend. As a result, research in the field of biodegradable and renewable materials is receiving increasing attention from researchers. Among these materials, polylactic acid (PLA) derived from renewable resources such as cassava and corn starch [1,2] exhibits the unique property of complete biodegradability into water and carbon dioxide under specific conditions. Furthermore, PLA can be processed into various forms using methods like injection molding, extrusion, and blow molding [3,4]. Hence, it is regarded as one of the most promising alternatives to petroleum-based materials and finds extensive applications in sectors including food packaging [5,6], biomedicine [7,8], and construction materials [9]. Nevertheless, PLA has its limitations, including poor thermal stability, insufficient toughness, and subpar moisture and oxygen barrier properties, which restrict its widespread application. These limitations restrict the use of pure PLA in certain conditions; for instance, brittleness may lead to issues related to strength and durability in some engineering applications, low barrier properties may impact the isolation performance of gases and moisture in packaging applications, and low heat resistance may limit its application in certain high-temperature conditions. Consequently, research on PLA modification has become a topic of central attention. This review provides an overview of the current methods for enhancing PLA properties, with a specific focus on research involving the reinforcement of PLA with modified biomass such as cellulose, lignin, and starch.

## 2. PLA

**Figure 1:**
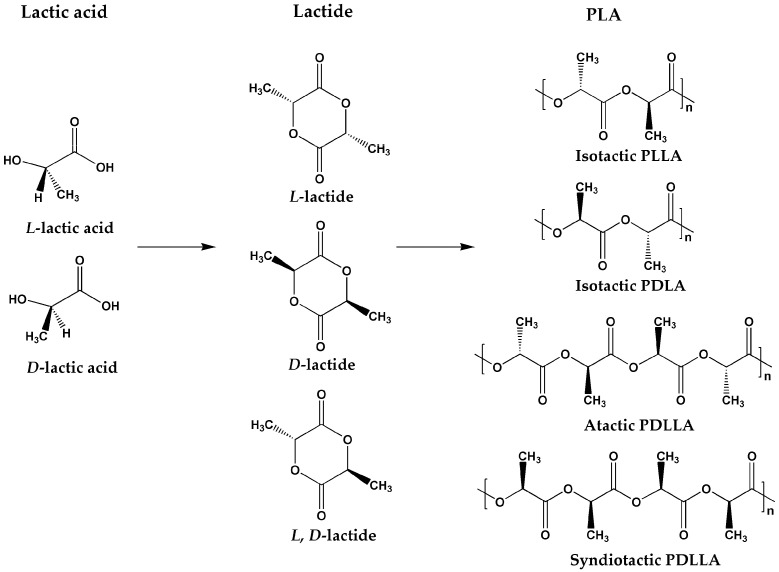
Stereoisomers of lactic acid, lactide, and PLA.

Polylactic acid (PLA) is a polymer primarily synthesized from lactic acid (LA). Because of the presence of asymmetric carbon atoms in lactic acid, two enantiomeric forms, L and D, exist [10,11], giving rise to L-lactide, D-lactide, and L, D-lactide. Consequently, different types of PLA can be produced in the process of synthesis of PLA (as shown in Figure 1): isotactic poly-L-lactic acid (PLLA), isotactic poly-D-lactic acid (PDLA), and atactic poly-L, D-lactic acid and syndiotactic poly-L, D-lactic acid (PDLLA) [12]. Therefore, the various distributions of stereoisomers within PLA chains have an impact on the physical properties, such as mechanical properties, barrier properties, and thermal stability.

### 2.1. Application of PLA

Currently, PLA is widely employed in various fields, including food packaging, medical supplies, and 3D printing [13]. In the field of food packaging, PLA is utilized to produce films, utensils, and food containers, with its biodegradability making it highly favored in the environmentally conscious market. In the medical field, PLA is applied in the manufacturing of disposable medical devices, medical fibers, etc., leveraging its distinctive biocompatibility and degradability, rendering it an ideal choice for medical applications. Furthermore, in the domain of 3D printing, as one of the common materials, PLA is highly favored due to its ease of processing and characteristics such as not generating harmful gases during processing.

While PLA possesses excellent biodegradability, renewability, and processability, it is simultaneously characterized by limitations such as brittleness, low heat resistance, and barrier properties. Therefore, to overcome these drawbacks, there is a current inclination towards the use of PLA composite materials. These materials consist of a PLA matrix and reinforcing materials. The commonly used reinforcing materials in the current market mainly include organic or inorganic micro/nanoparticles, short or long continuous fibers, and specific metal particles [14]. For example, introducing cellulose nanocrystals and Ag nanoparticles into PLA can effectively enhance the barrier properties of the composite material against oxygen and water vapor [15]. The addition of cellulose nanofibers significantly improves the toughness and mechanical properties of the composite material [16,17]. Additionally, composites with ZnO nanofibers exhibit excellent antibacterial activity and fatigue resistance, showing promising applications in the field of medical bone implant materials [18]. By incorporating appropriate reinforcing materials, PLA can further meet the increasing performance requirements in various fields.

### 2.2. Synthesis of PLA

**Figure 2:**
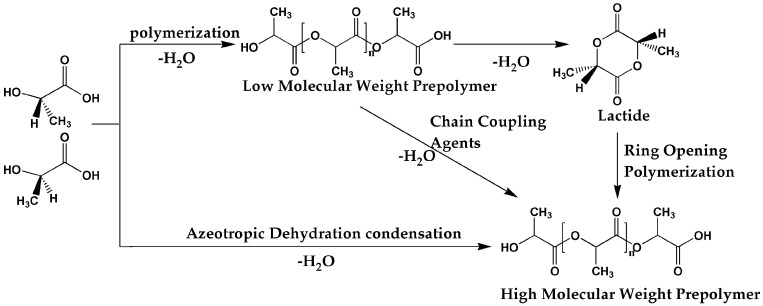
Different synthesis processes of PLA.

Figure 2 depicts the synthetic route of polylactic acid [19,20,21], which can be primarily categorized into three methods. The first method involves the direct condensation of lactic acid monomers. This process entails esterification reactions between the -COOH and -OH groups, linking lactic acid monomers together while removing water and alcohol from the reaction system. However, this method is associated with increasing viscosity of the mixture, making water removal challenging, resulting in the formation of low-molecular-weight products. Additionally, the uncontrolled structural features during polymerization can lead to inferior mechanical properties, limiting its application. To address the issue of low molecular weight in PLA synthesized by direct condensation, methods such as continuous melt condensation and the addition of chain extenders can be employed to increase PLA’s molecular weight [22]. Nevertheless, this approach requires precise reaction conditions and equipment, adding complexity and costs to the production process.

The second method involves the synthesis of PLA through the ring-opening polymerization of lactide, which is currently the primary industrial method for large-scale PLA production. The key steps in ring-opening polymerization are as follows [23]. Firstly, lactic acid undergoes dehydration to form oligomers, with low requirements for water removal due to the need for low-molecular-weight oligomers. Then, these oligomers are used to synthesize lactide, and, as the reaction is reversible, it requires control of reaction pressure and temperature to drive the equilibrium towards the formation of lactide. Finally, high-molecular-weight PLA is obtained through the ring-opening polymerization of lactide. In this process, the purification of lactide is a crucial step as insufficient purity hinders the synthesis of high-molecular-weight PLA. Currently, distillation is commonly employed for industrial purification of lactide, while, in laboratory settings, extraction and recrystallization methods are typically utilized. Now, the process of lactide ring-opening polymerization is well-established and considered the optimal method for producing high-molecular-weight PLA.

The last method involves co-boiling dehydration condensation to obtain PLA. In this process, lactic acid monomers undergo a polymerization reaction in an inert solvent. The water generated during the reaction can be separated using a co-boiling method, while the components in the co-boiling phase, including lactic acid and lactide, can be recycled back into the reactor to continue participating in the reaction, thus promoting the progression of the condensation reaction. However, this process requires many organic solvents, and there are issues related to the separation and purification of the products. Additionally, factors such as solvent evaporation and cooling during the reaction also increase energy consumption.

### 2.3. Enhancement of PLA

PLA has a glass transition temperature (*Tg*) of around 60 °C [24]. PLA exhibits poor thermal stability, inherent brittleness, limited toughness, and subpar moisture resistance, oxygen barrier properties, and hydrophilicity [25,26], which collectively restrict its widespread application. Consequently, addressing these limitations in PLA has emerged as a current research focal point. In recent years, approaches such as blending, copolymerization, and plasticization of PLA with other polymers [27,28] have proven effective in ameliorating some of its deficiencies, ultimately enhancing its overall properties and commercial viability.

As PLA is composed of lactic acid monomers, PLA-based copolymers with enhanced properties can be synthesized by copolymerizing lactic acid with other monomers [29]. For example, Luo et al. [30] copolymerized lactic acid with maleic anhydride (MAH) to synthesize a copolymer with double bonds, known as macromolecule polylactic acid-co-maleic anhydride (PLAM). PLAM copolymers exhibit increased flexibility compared to PLA, and their glass transition temperature (*Tg*) significantly decreases. Wang et al. [31] obtained polylactic acid–polyethylene glycol (PLEG) through the direct melt copolymerization of lactic acid and polyethylene glycol (PEG). Additionally, Luo et al. [32] synthesized copolymer polylactic acid-co-sorbitol [P(LA-co-SB)] using lactic acid and sorbitol (SB) as raw materials, and these copolymers all exhibit superior properties compared to PLA.

Plasticizers are widely employed polymer additives in industrial production, typically used to enhance the flexibility, extensibility, and processability of polymers [33,34]. Therefore, plasticization modification is a crucial approach for improving the mechanical properties of PLA. Presently, numerous studies have reported on effective plasticizers for enhancing PLA properties, including citrate esters [35], oxidized soya oil polymer (PSy-ox) [36], cinnamic acid (CA) [37], and epoxidized crude rubber seed oil (EcRSO) [38], all of which effectively enhance the mechanical properties of PLA. By increasing the flowability of PLA segments, these plasticizers augment the elongation at break and flexibility.

Mixing PLA with specific polymers through methods like direct melt blending to alter the type, composition, and subsequent processing of the blends is a means to produce blends with excellent strength and toughness, serving as a method to enhance the overall properties of PLA [39]. Currently, the PLA blending modification systems can be categorized into partial biodegradation and full biodegradation systems. The former are limited in application due to the presence of non-degradable polymer components. The latter refer to systems where all components in the PLA blend can fully biodegrade, offering a solution to contemporary environmental pollution issues. Such fully biodegradable systems hold great research prospects and application value. Typical fully biodegradable systems include biomass/PLA blends, which encompass components such as cellulose fibers from plant cell walls [40], hemicellulose [41], lignin [42], as well as chitin from the shells of crustaceans [43], starch, and natural substances like cyclodextrin [44].

In summary, PLA copolymerization, plasticization, blending modification, and their modification effects are outlined in Table 1. Among the modification methods for PLA, such as copolymerization and plasticization, blending PLA with specific polymers to address its limitations has been proven to be a more effective and economical approach. This method is straightforward and cost-effective. Currently, in the realm of PLA blending modifications, the PLA/biomass blending system has shown great promise. It not only effectively enhances PLA’s properties but also maintains the material’s biodegradability, establishing an entirely eco-friendly system. This contributes to alleviating environmental pollution issues and mitigating the problem of high PLA production costs.

## 3. Enhancement of PLA with Modified Biomass

Biomass is considered a green resource derived from renewable sources such as crop residues, bamboo, and other agricultural and forestry waste materials. It is produced through various physical, chemical, and biological processes, offering advantages like cost-effectiveness, wide availability, renewability, and environmental friendliness. With the growing emphasis on ecological conservation and sustainable resource utilization, biodegradable biomass materials have increasingly become the focus of widespread attention [45,46]. Blending biomass materials with PLA offers an effective solution to address the limitations of PLA, such as poor toughness, thermal stability, moisture, and oxygen barrier properties. However, within composite materials comprising biomass and PLA, many biomass materials contain highly polar hydroxyl groups, which can lead to inadequate compatibility at the interface with nonpolar polylactic acid. This incompatibility can impact the properties, structure, uniform dispersion, and interfacial strength of the composite materials. Therefore, improving the compatibility between hydrophilic biomass and hydrophobic PLA has been a perennial research focus. In the preparation of the majority of composite materials combining biomass with PLA, a common initial step involves modifying the biomass materials to enhance their interface compatibility with PLA, effectively addressing the drawbacks of PLA.

### 3.1. Enhancement of PLA with Modified Cellulose

**Figure 3:**
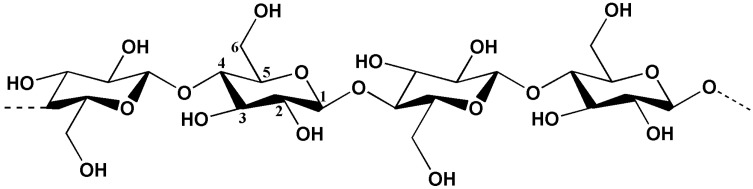
The chemical structure of cellulose.

Cellulose is a rich source of natural, renewable organic material found extensively in various forms of biomass. It is a green, cost-effective, and widely available natural fiber with excellent biocompatibility, biodegradability, and thermal stability, making it suitable for a wide range of applications, including textiles, food, chemicals, environmental solutions, and construction. The molecular structure of cellulose consists of linear high-polymer chains formed by D-glucose rings connected via *β*-1,4 glycosidic linkage. This regular structure, along with the abundance of hydroxyl groups, facilitates the formation of numerous hydrogen bonds between molecules [47,48,49] (Figure 3). These cellulose molecules aggregate in an ordered parallel layer within plant cell walls, algal cell walls, or bacterial structures, creating primary fibers that ensure structural rigidity and stability [50]. Currently, cellulose is extensively employed as a reinforcing agent in PLA composite materials. By incorporating various types of cellulose materials, including cellulose fibers and nanocellulose, it becomes possible to effectively modify multiple aspects of PLA properties, such as mechanical properties, thermal stability, barrier properties, crystalline characteristics, and more. Simultaneously, the inclusion of low-cost cellulose reduces PLA production costs, enhancing its commercial competitiveness.

#### 3.1.1. Mechanical Properties

Researchers have introduced modified nanocellulose (NC) into PLA to enhance its properties. The smaller particle size of nanocellulose, coupled with improved compatibility achieved through modification, offers unique advantages for enhancing PLA’s properties. Nanocellulose is a nanoscale cellulose material extracted from natural cellulose fibers, with at least one dimension measuring less than 100 nm. Incorporating nanocellulose into PLA through hydrogen bonding can enhance the interfacial interactions between the two, thereby improving the overall properties of PLA [51,52]. Nanocellulose is primarily categorized into three types [53,54,55]: (1) cellulose nanocrystals (CNC), also known as cellulose nanowhiskers (CNW) or nanocrystalline cellulose (NCC); (2) cellulose nanofibrils (CNF), also referred to as nanofibrillated cellulose (NFC); and (3) bacterial cellulose (BC). These three types of nanocellulose exhibit variations in morphology, size, and preparation methods, as detailed in Table 2.

The rigid structure of cellulose enables it to effectively enhance the mechanical properties of PLA when used as a filler. Niu et al. [56] prepared cellulose nanocrystals (CNC) through microcrystalline cellulose (MCC) and modified CNC with various fatty acid chlorides, including valeryl chloride, octanoyl chloride, dodecanoyl chloride, and stearoyl chloride, to obtain four types of aliphatic-chains-grafted cellulose nanocrystals (ECNCs). PLA/ECNCs composites were fabricated using a solvent casting method. The results revealed a significant improvement in the elongation at break and storage modulus of the composite material, both of which surpass those of pure PLA. This approach involves grafting fatty chains onto cellulose nanocrystals (CNC) to induce entanglement behavior with PLA, thereby disrupting the regular arrangement of PLA crystalline regions and forming a stable network structure. Simultaneously, with an increase in the length of fatty chains, esterification reactions gradually extend into the internal regions of CNC. The esterification layer progressively thickens, forming a corona structure. This ensures efficient flexibility and mobility of PLA molecular chains, leading to further enhancement in toughness.

In addition to the entanglement behavior, modifying the reinforcing materials can enhance their hydrophobicity to improve compatibility with PLA. This facilitates the formation of a dense network structure, resulting in superior composite materials. Long et al. [57] prepared formic-acid-modified formyl cellulose (FC) and PLA composite films using a solvent casting method. The hydroxy groups of cellulose reacted with formic acid to form ester groups, rendering FC more hydrophobic and facilitating its dispersion in the PLA matrix to form a network structure. This led to an improvement in the tensile strength and Young’s modulus of PLA. Directly blending polar reinforcing materials with nonpolar PLA may result in defects and voids between the two phases, ultimately weakening the mechanical properties of the composite material. Xu et al. [58] introduced polydopamine (PDA) grafting onto the surface of cellulose nanocrystals (CNC) to prepare graft-modified (PLA/CNC-PDA) composite materials. After introducing PDA grafting, the polarity of CNC decreased, and PDA acted as a bridge, enhancing the interfacial adhesion between CNC and PLA, reducing defects between them. As a result, the tensile strength and elongation at break of the composite film were higher than those of pure PLA film. The addition of modified cellulose contributed to the enhancement of PLA’s mechanical properties, as summarized in Table 3.

#### 3.1.2. Barrier Properties and Antimicrobial Properties

Cellulose enhances the mechanical properties of PLA, and, owing to its unique porous network structure, biodegradability, and excellent barrier properties, also renders it a superb material for food packaging applications. Furthermore, it can enhance PLA’s antimicrobial and barrier properties. Huang et al. [59] modified cellulose nanocrystals with methacrylamide (MAM) and cetyltrimethylammonium bromide (CTAB) to prepare PLA/modified cellulose composite materials (PLA-MC8 and PLA-CC8). The modified CNC forms a dense protective film on the surface of PLA, enhancing the barrier properties against water vapor and oxygen. Therefore, when 8% filler is added, both composite films exhibit a significant reduction in water vapor permeability (WVP) and oxygen transmission rate (OTR). In comparison to pure PLA films, PLA-CC8 shows a reduction of 50.8% in WVP and 69.6% in OTR values. Simultaneously, the composite film achieves a deactivation rate of 99.9% or more against Staphylococcus aureus and Escherichia coli, inhibiting the replication of bacterial cell DNA and demonstrating excellent antibacterial performance. 

Furthermore, esterification modification of cellulose involves grafting fatty chains onto its molecular chains, forming a hydrophobic structure that hinders the penetration of water vapor. Lafia-Araga et al. [60] modified cellulose nanofibers (CNF) by catalyzed lactic acid esterification and fabricated PLA composites with modified CNF. Following the treatment, the hydroxyl groups on the CNF surface decrease. Additionally, the presence of the fatty carbon chain of lactic acid inhibits the formation of water–nanofiber bonds, enhancing the barrier to water. The permeability of water vapor through composite films is reduced by approximately 20% compared to pure PLA films.

### 3.2. Enhancement of PLA with Modified Lignin

**Figure 4:**
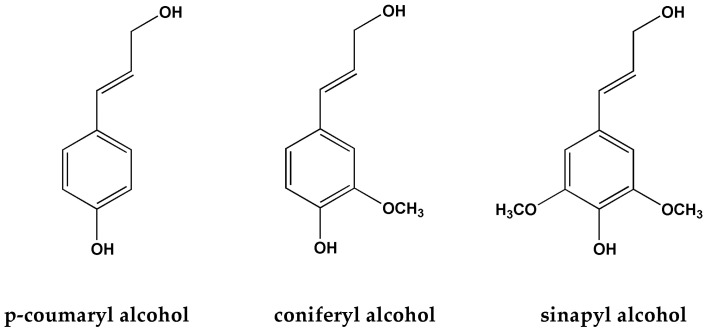
Three structural units of lignin.

**Figure 5:**
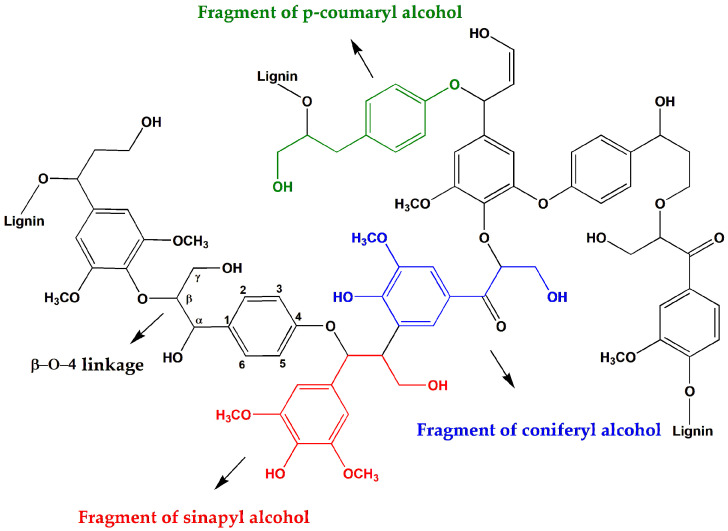
The main connections between lignin units.

Lignin is currently the sole non-petroleum resource in nature capable of providing renewable aromatic compounds. It is the world’s second-largest biomass resource, with storage capacity second only to cellulose, and is widely distributed in plant lignocellulosic tissues. Lignin in nature is often interconnected with cellulose, hemicellulose, and other components, forming lignocellulosic biomaterials, accounting for approximately 20–30% of the total biomass. Its chemical structure is composed of three basic alcohol monomers [61,62], such as p-hydroxyphenyl propane (H, derived from p-coumaric alcohol), guaiacyl propane (G, derived from coniferyl alcohol), and syringyl propane (S, derived from sinapyl alcohol), as shown in Figure 4. These three units are primarily connected by C–C and C–O linkages, forming a three-dimensional network of biopolymers (as shown in Figure 5). The most abundant bond in lignin is the *β*-O-4 linkage, accounting for more than 50% of the total. This linkage is formed by the phenolic hydroxyl and carbonyl reactions of the phenylpropane units, which are readily formed and provide lignin excellent structural stability and strong antioxidative properties. Additionally, lignin contains other bond types, such as *α*-O-4, 4-O-5, *β*-*β*, and *β*-5, although they are present in lower quantities. Consequently, due to its wide availability, biodegradability, excellent antimicrobial and antioxidative properties, as well as strong stability, lignin has attracted increasing attention in research. It has been successfully utilized to prepare functional composite materials, finding wide applications in areas such as medical health, 3D printing, and food packaging [63,64].

#### 3.2.1. Mechanical Properties

Due to the natural linkage of lignin with cellulose and hemicellulose, it requires separation through chemical or physical methods. Based on different separation methods, lignin can be mainly categorized into [65,66] Kraft lignin (KL), lignosulfonates (LS), soda lignin (SL), organosolv lignin (OL), and others. However, different separation methods yield lignin with different structures and chemical properties, as detailed in Table 4. The blending of lignin with PLA can lead to the development of renewable functional composite materials with improved properties. For instance, Esakkimuthu [67] modified Kraft lignin through etherification reactions, using oxypropylated Kraft lignin (OPKL) as raw materials to prepare PLA/OPKL composite materials. Lignin etherification involves the introduction of newly formed fatty hydroxyl groups through an oxypropylation process, reducing the original phenolic hydroxyl group count. The introduction of oxypropyl chains increases the homogenization of lignin in the system, thereby enhancing the overall plasticity. The composite material exhibits higher elongation at break and tensile strength compared to pure PLA, and the elongation at break increased by approximately 10% compared to pure PLA.

Simultaneously, the reduction in particle size also contributes to better compatibility between lignin and PLA. Makri et al. [68] prepared composite materials of microsized (diameter of 2.38 μm) and nanosized (diameter of 524 nm) lignin with PLA (PLA-L and PLA-NL) through melt blending; the reduction in particle size of lignin effectively addresses the issue of poor interface compatibility between PLA and lignin. Both types of composite materials exhibited higher tensile strength and Young’s modulus compared to pure PLA. In particular, nanosized lignin has smaller and more uniform particle size and exhibited superior mechanical properties. The maximum tensile strength of the composite materials PLA-L and PLA-NL is increased by approximately 138% and 184%, respectively, compared to pure PLA. Furthermore, the maximum Young’s modulus for both composites exhibits an elevation of approximately 115% as compared to pure PLA. Yan et al. [69] fabricated composite materials of PLA with esterified lignin (PLA/AML). Esterification replaces hydroxyl groups on lignin with ester and carboxyl groups, facilitating compatibility with PLA. The tensile strength and Young’s modulus of the composite material are slightly higher than those of pure PLA, with increases of 18.4% and 19.5%, respectively. The esterified composite material exhibits even more outstanding mechanical properties.

#### 3.2.2. Thermal Stability

Lignin possesses high thermal stability, characterized by the presence of composite phenylpropane units with stable aromatic structures. Some hydroxyl groups in lignin undergo resonance effects at elevated temperatures, enhancing the stability of its aromatic structures and impeding cleavage. Therefore, compounding lignin with PLA can enhance the thermal stability of PLA.

**Figure 6:**
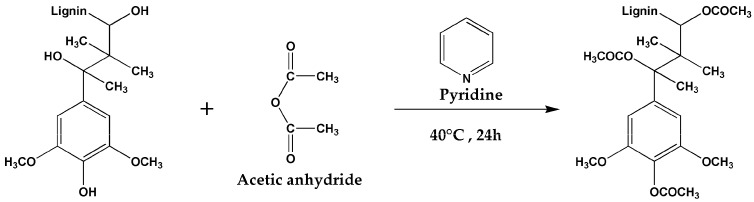
The process of lignin acetylation.

Gordobil et al. [70] utilized commercial alkali lignin (CL) as well as lignin extracted from almond shells (OL) as raw materials. They employed a twin-screw extrusion process to prepare acetylated (ACL and AOL) and non-acetylated PLA/lignin composites. The initial degradation temperature and maximum weight loss temperature of the composite material are both higher than those of pure PLA, demonstrating superior thermal stability. This is attributed to the presence of stable composite benzyl propane units in lignin, which imparts higher thermal stability and enhances the overall thermal stability of the composite material. Additionally, the decomposition temperature of the unmodified composite material is slightly higher than that of the acetylated one. It is speculated that, after the acetylation treatment of lignin, a significant number of the original hydroxyl groups were likely replaced by acetyl groups (as shown in Figure 6). As mentioned earlier, some individual hydroxyl groups may undergo resonance effects at high temperatures. However, after the acetylation treatment, these hydroxyl groups might be reduced, potentially leading to a minor decrease in thermal stability.

Cazacu et al. [71] modified lignosulfonate using a plasma method to prepare PLA/unmodified lignosulfonate (PLA/LA) and PLA/plasma-modified lignosulfonate (PLA/MLS) composite materials. In the presence of carboxylic acid modifiers, new functional groups are introduced to enhance compatibility with PLA. Compared to pure PLA, the initial decomposition temperature is slightly increased, indicating improved thermal stability of the composite material. Simultaneously, the cold crystallization temperature (*Tcc*) decreases, promoting the crystallization process of PLA. Notably, PLA/MLS exhibits a higher initial decomposition temperature, as summarized in Table 5, indicating an enhanced compatibility between plasma-modified lignosulfonate and PLA.

#### 3.2.3. Barrier Properties, UV Protection, and Antimicrobial Properties

Lignin molecules contain a significant number of functional groups, such as phenolic rings, ketone groups, and hydroxyl groups. These functional groups provide lignin with the capability to offer protection against ultraviolet (UV) radiation across the entire UV spectrum. Consequently, lignin exhibits a broad-spectrum UV shielding ability. Therefore, lignin holds great potential for applications in the field of food packaging. When employed as a food packaging material, exposure to sunlight, water vapor, and oxygen can lead to food deterioration. However, lignin boasts outstanding properties, including UV resistance, antioxidative capabilities, and antimicrobial characteristics.

Boarino et al. [72] employed a grafting method to prepare composite films of PLA-grafted lignin nanoparticles (PLA-LNPs) with PLA. In the 280 nm ultraviolet region, the UV transmittance of the composite material drops to below 10%, and, with the increase in filler ratio, it almost completely blocks the ultraviolet rays. This is attributed to the excellent UV blocking properties of lignin, whereas the UV transmittance of pure PLA film is approximately around 67%. After grafting treatment on lignin nanoparticles, most of the PLA grafts grow with aliphatic hydroxyl as initiators, leading to a significant reduction in the ratio of phenolic hydroxyl to aliphatic hydroxyl. This results in a uniform distribution in the PLA matrix. In untreated blends, phase separation and aggregation occur. Therefore, when water vapor passes through the film, the grafted composite material with a more tortuous path significantly reduces the water vapor permeability of the film. Buzarovska et al. [73] prepared PLA/alkaline lignin composite films (PLA/aL). A composite film containing 10% lignin exhibits antibacterial capabilities against Staphylococcus aureus more than ten times higher than pure PLA, along with a significant reduction in water vapor permeability. 

In addition to graft modification, acetylation is also a commonly used modification method. Kim et al. [74] prepared PLA/lignin (PLA/LIG) and PLA/acetylated lignin (PLA/a-LIG) films. Due to the inherent brown color of lignin, an increase in its content results in darker film color and reduced transparency. Therefore, within the visible light spectrum, composite films exhibit lower transparency compared to pure PLA films. However, under the same filler content, PLA/a-LIG films demonstrate superior light transmittance to PLA/LIG films. This is attributed to acetylation, where hydrophilic hydroxyl groups are replaced with hydrophobic acetyl groups, converting hydrophilic lignin into hydrophobic lignin. This process reduces the hydrogen bond strength within lignin molecules and decreases the structural domain size of lignin aggregation when mixed with organic polymers. As a result, it enhances compatibility with PLA, leading to higher light transmittance. Moreover, composite films achieve ultraviolet blocking rates of over 97.5%, significantly surpassing the 5% of pure PLA films.

### 3.3. Enhancement of PLA with Modified Starch

**Figure 7:**
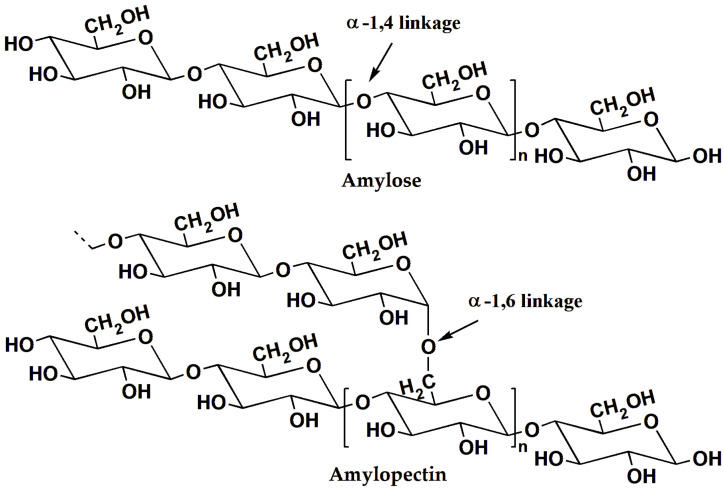
The structures of amylose and amylopectin.

Starch is a natural high-molecular-weight material like cellulose and lignin, and is composed of repeating glucose units linked by glycosidic bonds. It is widely available, cost-effective, and environmentally friendly. Natural starch comprises two main types of macromolecules [75], namely amylose and amylopectin (as illustrated in Figure 7). Amylose is primarily linked through *α*-1,4 glycosidic bonds, and its molecular structure is relatively simple, allowing it to be soluble in water. In contrast, amylopectin contains *α*-1,6 glycosidic bonds, resulting in a more complex molecular structure that is insoluble in water. Consequently, different sources of starch exhibit varying ratios of amylose and amylopectin, leading to differences in their properties. Starch-based materials are conducive to film formation, yielding films with excellent gas barrier properties, flexibility, non-toxicity, and taste neutrality. Blending starch with PLA not only effectively reinforces PLA but also offers significant potential for applications in the food packaging and biomedical fields [76]. However, due to the poor compatibility between hydrophilic starch and hydrophobic PLA, modification is necessary prior to their blending to enhance the interfacial compatibility of the two components.

#### 3.3.1. Mechanical Properties

Compounding PLA with modified starch enhances mechanical properties. Jariyasakoolroj et al. [77] prepared three different silane-modified starches for the production of PLA/silane-modified starch composites. Among them, the PLA/silane-modified starch material (PLA/CP-starch) modified with 3-chloropropyl trimethoxysilane (CPMS) exhibited the most outstanding mechanical properties. Starch exhibits a robust network of intermolecular and intramolecular hydrogen bonds. Following modification, the hydrogen bonds between starch molecules are disrupted, and a crosslinked network is formed between starch chains through silane–alcohol bonds, resulting in increased hydrophobicity. The silanol groups on silane-modified starch react with PLA to form covalent bonds, thereby promoting compatibility. The composite film showed higher tensile strength, elongation at break, and Young’s modulus compared to pure PLA film, and the tensile strength increased by 13.8% compared to the pure PLA film.

Xiong et al. [78] employed castor oil (CO) and hexamethylene diisocyanate (HDI) for the graft modification of starch to prepare blends with PLA/CO/HDI–graft-starch composites. The impact strength and elongation at break of composite films are approximately increased by 128% and 1260% compared to pure PLA, demonstrating excellent toughness because, when HDI is grafted onto starch particles, the hydroxyl groups on CO react with the isocyanate groups on the grafted starch (HGST), causing CO molecules to accumulate on the starch particles, forming a flexible interface. Simultaneously, excess isocyanate groups react with the terminal carboxyl groups (COOH) or hydroxyl groups on PLA, resulting in the formation of a compatible blend of PLA, HGST, and CO. Additionally, another study was conducted in which the surface of starch was hydrophobically modified with two types of epoxy resins [79]. This resulted in the preparation of PLA/starch and PLA/modified starch composite materials. The PLA/modified starch composite materials exhibited higher tensile strength, tensile modulus, and elongation at break compared to PLA/starch composite materials, especially in terms of tensile modulus, which exceeded that of pure PLA film.

#### 3.3.2. Barrier Properties

The composite film of PLA and modified starch exhibits excellent barrier properties. Kulkarni et al. [80] used maleic anhydride (MA) to promote the grafting of glycerol onto starch to obtain maleic anhydride thermoplastic starch (MTPS), and prepared PLA/MTPS composite materials. The water vapor permeability (WVP) and oxygen permeability (OP) of composite films are significantly lower than those of pure PLA because the high oxygen barrier of starch may contribute to reducing the oxygen permeability (OP) of the blend film. Additionally, the increased crystallinity of PLA results in the formation of non-permeable regions within the crystalline structure, creating a tortuous path for the diffusion of permeants. As the MTPS ratio increases, the reduction in WVP and OP becomes progressively smaller. This could be due to the fact that the average particle size of composite materials gradually increases, and smaller particles are beneficial for forming more tortuous pathways on the film surface, thereby improving the barrier properties against water vapor and oxygen. Additionally, Xu et al. [81] prepared graphene oxide (GO)-modified starch and combined it with PLA to produce PLA/GO@starch composite. The moisture and oxygen barrier properties were notably enhanced, as indicated in Table 6. The oxygen permeability of composite films significantly decreased. From the above, we can conclude that incorporating starch into the PLA enhances its barrier properties. Furthermore, when modified starch is subsequently blended with PLA, it can lead to a more uniform dispersion of starch in PLA. This leads to a more uniform dispersion of starch in PLA, forming a dense barrier layer and more effectively strengthening the barrier properties.

## 4. Biomass Modification Methods

In summary of the modified biomass-reinforced PLA presented above, it is evident that these modified biomasses have effectively mitigated some of the shortcomings of PLA, including its thermal stability, toughness, and barrier properties. This has substantially broadened the scope of PLA applications. Among the mentioned cellulose, lignin, and starch, each biomass type contains a wealth of hydroxyl groups, making them highly hydrophilic. However, in the case of direct blending with the hydrophobic PLA, their interface compatibility is comparatively poor. This can result in non-uniform dispersion in the PLA matrix, potentially diminishing PLA’s original properties and thus failing to achieve the intended reinforcement. Consequently, the modification of these biomasses to enhance their hydrophobicity and improve compatibility with PLA is necessary. Currently, modification methods for these biomasses primarily fall into two categories: physical and chemical modifications. Physical methods encompass techniques such as nanoscaling and plasma treatments, while chemical methods include esterification, amidation, and polymer grafting, among others. After achieving significant advancements in enhancing its toughness and heat resistance, modified PLA demonstrates unique advantages compared to other commonly used biodegradable materials, such as polybutylene succinate (PBS) and poly (butylene adipate-co-terephthalate) (PBAT). The modified PLA demonstrates comparable toughness and heat resistance when compared to PBS and PBAT. However, PLA holds a superior advantage in terms of raw material sourcing. PBS originates from petroleum or natural gas, and PBAT’s raw materials differ, deriving from synthetic polyesters and polyamides. In contrast, PLA’s raw materials are derived from renewable biomass, providing a more extensive and sustainable foundation. Additionally, PLA exhibits excellent transparency, enhancing its commercial value.

### 4.1. Plasma Modification

**Figure 8:**
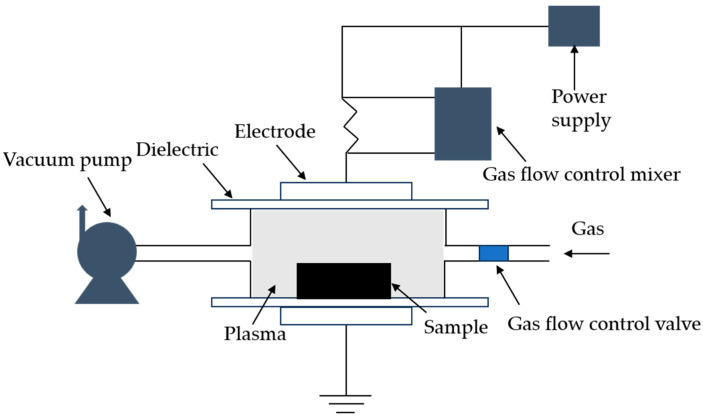
Schematic diagram of the plasma modification treatment equipment.

Among the modification methods, plasma treatment stands out as a safe, environmentally friendly, and convenient approach. In comparison, nanomaterial-based modification methods incur higher production costs, while chemical modifications like esterification, amidation, and polymer grafting involve toxic reagents and are prone to causing environmental pollution. Plasma primarily consists of ions (positive and negative), free radicals, electrons, and excited or ground-state atoms [82,83]. By supplying sufficient energy through external sources such as voltage application and irradiation, a plasma is generated within a reactor (as depicted in Figure 8). This method boasts numerous advantages, including swiftness, robust functionality, eco-friendliness, and cost-effectiveness. Plasmas can be categorized based on temperature into high-temperature plasmas and low-temperature plasmas. The gases used in plasma modification can be air, noble gases, or gases produced through the vaporization of organic solvents. They may also be a mixture of various types of gases. Once gas undergoes plasma treatment, it interacts with the surface of the biomaterial fibers, thereby achieving plasma-based modification.

Plasma modification of biomasses primarily involves two approaches: one involves surface etching, resulting in surface roughening of the material, accompanied by bond breakage and the formation of new radicals, thereby increasing surface roughness and water contact angle; the other approach involves the introduction of functional groups. Through plasma treatment, the intermediates and radicals can react with functional groups on biomasses, leading to the grafting of hydrophobic moieties on the fiber surface. Both of these methods can be employed to enhance the hydrophobicity of biomasses and improve their compatibility with PLA. Jang et al. [84] conducted plasma treatment on the surface of coconut fibers and prepared coconut fiber/PLA composite materials using a blending and spinning method. Plasma treatment introduces chemical groups such as carboxyl and ester onto coconut fibers, enhancing the compatibility of the interface with PLA. In comparison to the untreated counterpart, the plasma-treated composite material shows improvements in tensile strength, Young’s modulus, and elongation at break. Siro et al. [85] treated nanofibrillated cellulose (NFC) with plasma in a mixture of tetrafluoromethane (CF_4_) and oxygen (O_2_) gases. Some hydroxyl groups of cellulose are carboxymethylated (-CH_2_COOH), resulting in a reduction in hydrophilic functional groups. The water contact angle of NFC increased to approximately 68.8 ± 16.9°, approaching that of PLA (75–80°), thereby enhancing the adhesion between the two materials and further improving the performance of PLA.

### 4.2. Mechanisms of Plasma Treatment on Biomass 

As previously mentioned, the molecular structure of cellulose consists of D-glucose units linked by β-1,4 glycosidic bonds to form a polymer. Natural cellulose comprises crystalline and amorphous regions. During plasma treatment, highly reactive ions such as ∙O^2−^ and ∙OH, exhibiting strong oxidative properties, selectively attack the disordered amorphous regions of cellulose. This leads to a reduction in the length of cellulose molecular chains and a decrease in polymerization degree. In contrast, the ordered arrangement and stable structure of cellulose in the crystalline regions, characterized by numerous hydrogen bonds, remain relatively unaffected by plasma treatment. While the primary molecular structure of cellulose is challenging to disrupt, plasma treatment enhances the reactivity of cellulose reactions.

In the process of plasma treatment of lignin, lignin, composed of three phenylpropane monomers based on alcohol units, forms a three-dimensional biopolymer through C–C and C–O linkages, creating a complex molecular structure. Plasma treatment induces the cleavage of molecular bonds in lignin, generating radical intermediates. These radical intermediates undergo coupling reactions [86], resulting in the formation of novel molecular structures and an augmentation of the reactivity of lignin. Simultaneously, the hydroxyl functional groups of lignin undergo alterations during plasma treatment. Changes in these functional groups have the potential to impact the hydrophilicity, surface properties, and interactions with other substances of lignin. In summary, plasma treatment, by inciting chemical reactions within biomass and inducing alterations in molecular structure, facilitates its subsequent conversion and utilization.

Through the application of these methods to modify biomasses, effective enhancement of interface compatibility with PLA is achieved, enabling uniform dispersion within the PLA matrix, and ultimately reinforcing the PLA. The specific applications of each modification method are summarized in Table 7.

## 5. Conclusions and Outlook

The most critical challenge in reinforcing PLA with biomasses is addressing the compatibility issues between the hydrophilic biomass and the hydrophobic PLA. Unmodified biomass exhibits poor dispersion within the PLA matrix and weak interfacial adhesion, failing to achieve the desired reinforcement effect. Therefore, it is essential to subject the biomass materials to appropriate pretreatments, such as size reduction, introduction of functional groups, and polymer grafting, before blending them with PLA. Various modification methods yield different results, enhancing the compatibility of these two components. PLA has its limitations, including poor thermal stability, insufficient toughness, and subpar moisture and oxygen barrier properties. These modifications target specific drawbacks of PLA rather than seeking comprehensive improvement, thus offering valuable insights into enhancing PLA through biomass reinforcement. In the aforementioned context, we have learned that blending modified biomass with PLA can selectively enhance certain drawbacks of PLA. For instance, aliphatic chains-grafted cellulose nanocrystals can increase the elongation at break of PLA by more than six times, while formylated cellulose enhances the Young’s modulus of PLA by over four times, effectively addressing the brittleness inherent in PLA. Modified lignin contributes to the improvement of PLA’s thermal stability, elevating its initial decomposition temperature by over 50 °C and imparting excellent UV shielding properties. Furthermore, post-modification of starch proves effective in enhancing PLA’s barrier properties against water vapor and oxygen, with graphene-oxide-modified starch approximately reducing PLA’s oxygen permeability to 11%. Such research aims to address the inherent limitations of PLA, enhance its commercial value, and explore broader applications for PLA in various fields.

## Figures and Tables

**Table 1:** Summary of PLA modifications.

Modification Method	Treatment or Added Material	Effect	Ref.
Copolymerization	PLAM: lactic acid (LA) and maleic anhydride (MAH)	*T*_g_ decreased to 2.3 °C and increase in flexibility *T*_m_ decreased from 120 °C to 96.7 °C	[30]
	PLEG: lactic acid (LA) and polyethylene glycol (PEG)	*T*_g_ decreased to 20.7–33.6 °C and increase in flexibility Improving hydrophilicity, and the contact angle is 59.9°–68.7°	[31]
	P(LA-co-SB): lactic acid (LA) and sorbitol (SB)	Decrease in crystallinity and *T*_g_ decreased to 30–49 °C	[32]
Plasticization	Triethyl citrate (TEC) Acetyl tributyl citrate (ATBC)	*T*_g_ decreased to 10.29 °C and 12.21 °C Decrease in storage modulus and increase in flexibility and mobility Decrease in *T*_m_ and thermal stability	[35]
	Oxidized soya oil polymer (PSy-ox)	Increasing the elongation by 10 times *T*_g_ decreased to 17–20 °C and increase in plasticity Decrease in tensile strength	[36]
	Cinnamic acid (CA)	Thermal resistance is improved, decomposition temperature is increased by more than 40 °C Decrease in *T*_g_ and increase in plasticity Improving the mechanical properties and reducing the elastic modulus Increase in the water barrier properties and oxygen barrier properties	[37]
	Epoxidized crude rubber seed oil (EcRSO)	Improvement of 815% in elongation at break and 1370% in tensile toughness Improving the thermal stability, and the initial weight loss temperature is increased by 50.15 °C	[38]
Blending	PLA/α-Cellulose	Improving the elongation at break and impact strength Increase in crystallinity	[40]
	PLA/Lignin-containing cellulose nanofibrils (LCNFs)	Improving the tensile strength by 37% and tensile modulus by 61.1% Increase in the thermal stability *T*_g_ decreased to 52.6 °C and increase in flexibility	[42]
	PLA/Chitin	Improvement of 275% in tensile strength and 13% in tensile modulus	[43]
	PLA/Starch	Increase in the oxygen barrier properties Increase in tensile strength and Young’s modulus	[44]

**Table 2:** Summary of structural characteristics of different nanocellulose types.

NC Type	Diameter (nm)	Length [55] (um)	Shape	Source Material	Synthesis Method
CNC	4–70	0.05–0.5	Acicular, short rodlike	Wood, cotton, etc.	Acid hydrolysis
CNF	5–100	0.5–2	Filiform, mesh	Wood, potato tubers, etc.	Mechanical
BC	20–100	>1	Ribbon	Glucose, ethanol, etc.	Microbial reaction

**Table 3:** Effect of modified cellulose on mechanical properties of PLA.

Matrix	Filler	Mechanical Properties (Pristine Matrix: 100%)			Ref.
		Tensile Strength	Elongation at Break	Young’s Modulus	
PLA (7032D)	EOCNC (1%)	<75%	675%	-	[56]
PLA (Revode 190)	FC (1%)	148.6%	<70%	446%	[57]
PLA (4032D)	CNC-PDA (1%)	117.3%	133.0%	<100%	[58]

**Table 4:** Properties of different types of lignins and their separation processes.

Type of Lignin	Mn(g/mol)	Glass Transition Temperature (°C)	Separation Process
Kraft lignin [62]	1000–3000	100–150	Na_2_S, NaOH
Lignosulfonates [65]	15,000–50,000	120–140	HSO_3_^−^, Ca^2+^, Na^+^, etc.
Soda lignin [66]	800–3000	140–160	NaOH
Organosolv lignin [66]	500–5000	90–110	Formic acid, ethanol, water, etc.

**Table 5:** Effect of modified lignin on thermal properties of PLA.

Matrix	Filler	The Initial Degradation Temperature (*T_5%_*)/°C	The Maximum Weight Loss Temperature (*T_max_*)/°C	Ref.
PLA (3051D)	-	264	314	[70]
PLA (3051D)	ACL (5%)	325	360	
PLA (3051D)	AOL (5%)	318	365	
PLA (2002D)	-	321.1	376.7	[71]
PLA (2002D)	MLS (7%)	330.4	364.5	

**Table 6:** Effect of modified starch on the barrier properties of PLA.

Matrix	Filler	Barrier Properties (Pristine Matrix: 100%)		Ref.
		Water Vapor Permeability (WVP)	Oxygen Permeability (OP)	
PLA (3001D)	MTPS (1%)	<100%	73%	[80]
PLA	Starch (30%)	-	60.9%	[81]
PLA	GO@ starch (30%)	-	11.2%	

**Table 7:** Applications of different modification methods.

Modification Method	Matrix	Filler	Mechanical Properties (Pristine Matrix: 100%)		Barrier Properties (Pristine Matrix: 100%)		Ref.
			Tensile Strength	Young Modulus	Water Vapor Permeability (WVP)	Oxygen Permeability (OP)	
Nanometer	PLA (2003D)	CNC I (1%)	134%	145%	62.0%	31.1%	[87]
	PLA (2003D)	CNC II (1%)	172%	110%	68.8%	36.4%	[87]
Plasma modification	PLA (2002D)	LS (7%)	97.6%	117.5%	-	-	[71]
	PLA (2002D)	LS-OA (7%)	108.7%	129.4%	-	-	[71]
Esterification	PLA (3052D)	CNC (3%)	<100%	<100%	>46%	>45%	[88]
	PLA (3052D)	Cin-CNC (3%)	170%	137%	46%	45%	[88]
Amidation	PLA (Revode 190)	FC (1%)	148.6%	446%	59.4%	-	[57]
Graft	PLA (4032D)	CNCs (0.5%)	107.5%	104.6%	-	78.3%	[89]
	PLA (4032D)	CNCs-PEG (0.5%)	120.8%	113.9%	-	33.6%	[89]

CNC I: cellulose nanocrystals by acid hydrolysis; CNC II: cellulose nanocrystals by alkali treatment; LS: ammonium lignosulfonate; LS-OA: oleic-acid-modified ammonium lignosulfonate by plasma method; CNC: cellulose nanocrystals; Cin-CNC: cinnamate-modified cellulose; FC: formyl cellulose; CNCs: cellulose nanocrystals; CNCs-PEG: grafted polyethylene glycol on CNCs.

## Data Availability

Not applicable.
